# A new human chromogranin 'A' immunoradiometric assay for the diagnosis of neuroendocrine tumours

**DOI:** 10.1054/bjoc.2000.1659

**Published:** 2001-03

**Authors:** G P Bernini, A Moretti, M Ferdeghini, S Ricci, C Letizia, E D'Erasmo, G F Argenio, A Salvetti

**Affiliations:** Department of Internal Medicine and of; Oncology, University of Pisa, Pisa; Department of Clinical Sciences, University of Rome, Rome, Italy

**Keywords:** chromogranin A, phaeochromocytoma, neuroendocrine tumours

## Abstract

We investigated whether plasma chromogranin A (CgA), measured by a new immunoradiometric assay, may be a sensitive and specific marker of phaeochromocytoma and of other neuroendocrine tumours. This study involved 121 patients of whom 20 with phaeochromocytoma, 28 with other neuroendocrine tumours (19 gastroenteropancreatic tumors, 3 medullary thyroid and 6 small cell lung carcinomas), 25 with solid nonfunctioning adrenocortical tumours and 48 with essential hypertension. In addition, 130 normal subjects were taken as controls. Plasma catecholamines were measured by using high-performance liquid chromatography, and CgA by a two-site sandwich immunoradiometric assay involving monoclonal antibodies raised against the unprocessed central domain (145–245) of human CgA. Plasma CgA in controls (49.0 ± 3.1 ng ml^–1^, mean ± SE) and in essential hypertensives (50.8 ± 3.5 ng ml^–1^) was lower (*P*< 0.0001) than in adrenocortical tumours (91.8 ± 13.2 ng ml^–1^), in phaeochromocytomas (254 ± 49 ng ml^–1^) and in patients with other neuroendocrine tumours (469 ± 84 ng ml^–1^). Plasma CgA and catecholamines identified 13 and 18 out of 20 phaeochromocytomas with sensitivity of 65% and 90%, respectively. Combined measurement of both markers improved sensitivity up to 100%. In the other neuroendocrine tumours, CgA was abnormal in 23/28 cases (sensitivity 82%) and in 6 it was the only circulating marker of disease. In gastroenteropancreatic tumours, CgA measurement identified all cases (sensitivity 100%). Specificity of CgA in patients with essential hypertension was 98%. In conclusion, CgA determination showed high sensitivity in identifying gastroenteropancreatic tumours and, in association with catecholamines, in detecting patients with phaeochromocytoma. CgA sometimes appeared to be the only circulating marker of disease. Since the specificity of CgA proved to be excellent, this assay may be useful for diagnosis both of functioning and non-functioning neuroendocrine tumours. © 2001 Cancer Research Campaign http://www.bjcancer.com


British Journal of Cancer (2001) **84**(5), 636–642
© 2001 Cancer Research Campaign
doi: 10.1054/ bjoc.2000.1659, available online at http://www.idealibrary.com on IDEAL

http://www.bjcancer.com

### A new human chromogranin 'A' immunoradiometric assay for the diagnosis of neuroendocrine tumours

**GP Bernini[1], A Moretti[1], M Ferdeghini[2], S Ricci[2], C Letizia[3], E D'Erasmo[3], GF Argenio[1] and A Salvetti[1]**

[1]Department of Internal Medicine and of [2]Oncology, University of Pisa, Pisa; [3]Department of Clinical Sciences, University of Rome, Rome, Italy

**Summary** We investigated whether plasma chromogranin A (CgA), measured by a new immunoradiometric assay, may be a sensitive and specific marker of phaeochromocytoma and of other neuroendocrine tumours. This study involved 121 patients of whom 20 with phaeochromocytoma, 28 with other neuroendocrine tumours (19 gastroenteropancreatic tumors, 3 medullary thyroid and 6 small cell lung carcinomas), 25 with solid nonfunctioning adrenocortical tumours and 48 with essential hypertension. In addition, 130 normal subjects were taken as controls. Plasma catecholamines were measured by using high-performance liquid chromatography, and CgA by a two-site sandwich immunoradiometric assay involving monoclonal antibodies raised against the unprocessed central domain (145–245) of human CgA. Plasma CgA in controls (49.0 ± 3.1 ng $ml^{-1}$, mean ± SE) and in essential hypertensives (50.8 ± 3.5 ng $ml^{-1}$) was lower ($P < 0.0001$) than in adrenocortical tumours (91.8 ± 13.2 ng $ml^{-1}$), in phaeochromocytomas (254 ± 49 ng $ml^{-1}$) and in patients with other neuroendocrine tumours (469 ± 84 ng $ml^{-1}$). Plasma CgA and catecholamines identified 13 and 18 out of 20 phaeochromocytomas with sensitivity of 65% and 90%, respectively. Combined measurement of both markers improved sensitivity up to 100%. In the other neuroendocrine tumours, CgA was abnormal in 23/28 cases (sensitivity 82%) and in 6 it was the only circulating marker of disease. In gastroenteropancreatic tumours, CgA measurement identified all cases (sensitivity 100%). Specificity of CgA in patients with essential hypertension was 98%. In conclusion, CgA determination showed high sensitivity in identifying gastroenteropancreatic tumours and, in association with catecholamines, in detecting patients with phaeochromocytoma. CgA sometimes appeared to be the only circulating marker of disease. Since the specificity of CgA proved to be excellent, this assay may be useful for diagnosis both of functioning and non-functioning neuroendocrine tumours. © 2001 Cancer Research Campaign http://www.bjcancer.com

**Keywords**: chromogranin A; phaeochromocytoma; neuroendocrine tumours

Human chromogranin A (CgA), a 48-kDa protein encompassing 439 amino acids, belongs to the granin family and is widely distributed in secretory granules of endocrine and neuroendocrine cells (Konecki et al, 1987; Simon and Annis, 1989; Cetin and Grube, 1991).

CgA, secreted with the co-resident hormones, contains multiple dibasic sites important for proteolytic processing occurring at intragranular and extracellular levels. CgA thus behaves as a prohormone generating several bioactive peptides exerting intracrine, autocrine, paracrine and endocrine effects (Barbosa et al, 1991; Metz-Boutigue et al, 1993; Helle and Angeletti, 1994; Strub et al, 1996).

CgA, being co-stored and co-released with native hormones, is regarded as a useful tissue marker for a variety of neuroendocrine cells (Weiler et al, 1987; Deftos et al, 1988; Totsch et al, 1992; Rosa and Gerdes, 1994) and a possible sensitive circulating marker of neuroendocrine tumours (NET). Indeed high plasma CgA levels in patients with endocrine and neuroendocrine tumours, including phaeochromocytoma, have been reported (O'Connor and Bernstein, 1984; O'Connor et al, 1984; O'Connor and Deftos, 1986; Sobol et al, 1986; Hsiao et al, 1990a; Hsiao et al, 1990b; Deftos, 1991; Grondal et al, 1991; Hsiao et al, 1991; Johnson et al, 1993; Canale and Bravo, 1994; Kimura et al, 1997; Stridsberg and Husebye, 1997). In addition, CgA seems to reflect the secretory activity and burden of NET (Hsiao et al, 1990b; Grondal et al, 1991; Hsiao et al, 1991) and sometimes appears to be the only secretory product of hormone-negative endocrine (Sobol et al, 1989) and non-endocrine tumours (Deftos, 1991; Baudin et al, 1998).

Detection of the CgA antigen may therefore be of great clinical interest in differential diagnosis of suspected secretory tumours and in their follow-up. Circulating CgA was measured in earlier studies by several competition assays with different results (O'Connor and Bernstein, 1984; Dillen et al, 1989). However, since CgA is exposed to intensive proteolytic activity, sandwich methods involving monoclonal or polyclonal antibodies were subsequently developed (Bender et al, 1992; Syversen et al, 1994; Corti et al, 1996). Recently, Degorce et al (1999) introduced a two-site sandwich immunoradiometric assay with monoclonal antibodies directed against the median part of the protein, less exposed to proteolysis. By this method, Baudin et al (1998) showed that measurement of circulating CgA is a good marker for neuroendocrine tumours without highly conserved eutopic secretions, while its validity for phaeochromocytomas was still to be proven.

In the present paper we investigated whether plasma CgA, as evaluated by this new assay, is a predictor of phaeochromocytoma and of other-NET. In addition, we explored the possibility that CgA represents the only secretory product of apparently nonfunctioning solid adrenocortical tumours.

*Received 18 September 2000*
*Revised 23 November 2000*
*Accepted 29 November 2000*

*Correspondence to:* GP Bernini

### MATERIAL AND METHODS

### Subjects

121 patients (63 males, 58 females; mean ± SD age 49.8 ± 16.8 years; range 14–79 years) were enrolled. 48 had NET: 20 phaeochromocytoma, 3 medullary thyroid carcinoma, 6 small cell lung carcinoma and 19 gastroenteropancreatic tumours. 48 patients had essential hypertension and 25 patients had solid nonfunctioning adrenocortical tumours. Finally, 130 normal subjects were taken as controls. Demographic and clinical data of controls and of all patients are given in Table 1.

Essential hypertension, defined as recently reported (JNC VI), was confirmed after exclusion of all forms of secondary hypertension. In addition, in no case did CT or NMR show adrenal or abdominal masses. To further strengthen the diagnosis, these patients had been followed-up for at least 2 years and their blood pressure appeared to be well controlled with conventional antihypertensive therapy.

In patients with phaeochromocytoma, diagnosis was based on high plasma and urinary catecholamines or abnormal response to the glucagon test, and presence of adrenal or abdominal masses on CT or NMR (mean size 4.42 cm, range 2–7 cm). In 6 cases, metaiodobenzylguanidine scintigraphy further confirmed the diagnosis. Phaeochromocytomas were located at the adrenal level in all cases except one (para-aortic paraganglia). 15 phaeochromocytomas were sporadic and 5 familial (4 patients had multiple endocrine neoplasia type II, 1 had neurofibromatosis). In all patients diagnosis was confirmed by pathological analysis after surgery. Humoral follow-up and imaging data revealed 18 benign and 2 malignant tumours.

In patients with other-NET, diagnosis was established on the basis of clinical signs and symptoms, conventional imaging methods (CT, somatostatin receptor scintigraphy, angiography) and abnormal specific humoral markers (calcitonin, neuron-specific enolase, pancreatic polipeptide, serotonin, gastrin, carcinoembryonic antigen, α-subunit of glycoprotein, IGF-1). In all cases diagnosis was histologically confirmed. Gastroenteropancreatic tumours were classified according to their embryological origin: 10 patients had foregut-derived tumours (1 duodenum and 9 pancreas) and 9 had midgut-derived tumours (ileum and right colon).

Evaluation of patients with adrenocortical tumours was as follows: the majority ($n$ = 19) were discovered in hypertensive patients during routine analysis to exclude secondary forms of hypertension. 6 tumours were incidentally found in normotensive patients after abdominal ultrasound performed for hepatic ($n$ = 4) or genito-urinary disorders ($n$ = 2). In all cases, CT ($n$ = 23) or NMR ($n$ = 2) showed solid masses (18 unilateral and 7 bilateral) with size ranging from 10 to 40 mm (23.8 ± 1.6 mm, mean ± SE) and with apparently benign features. The hormonal study excluded abnormal activity of any of the following: adrenal medulla (basal plasma and urinary catecholamines and plasma catecholamines after 1 mg glucagon injection, when indicated), renin-angiotensin-aldosterone system (urinary aldosterone and plasma renin activity and aldosterone in upright position), glucocorticoids (urinary free cortisol, plasma cortisol before and after dexamethasone, plasma ACTH), androgens (free and total testosterone) and steroid precursors (plasma dehydroepiandrosterone sulphate, androstenedione and 17-OH-progesterone). When hormonal values resulted to be borderline an adrenal scintigraphic evaluation (metaiodobenzylguanidine- or 131-I norcholesterol-scintigraphy) was also requested.

The exclusion criterion for this study was renal failure with plasma creatinine >133.6 μM $l^{-1}$ (1.5 mg $dl^{-1}$) in order to avoid the well-known false positive results of CgA in this disease.

### Experimental design

All patients gave their informed consent to the study, which was approved by the local Ethical Committee. Patients taking drugs observed a pharmacological wash-out for at least 2 weeks prior to the study. However, 11 patients maintained antihypertensive therapy because of severe hypertension and/or target-organ damage, 4 were taking insulin or oral antidiabetics, 2 were on H2-antagonists and 2 on peripheral opioid-agonists. No patient with other-NET had started chemotherapy or somatostatin analogues prior to the study. All subjects maintained their usual diet with controlled salt intake (80–100 mEq $day^{-1}$ sodium and 60–80 mEq $day^{-1}$ potassium).

Tests were performed in the morning (08.00–09.00 h), after overnight fasting and in supine position. An indwelling 21-gauge scalp vein needle inserted in an antecubital vein was used for blood sampling. Controls underwent a blood sample after 30 minutes for measurement of plasma CgA and creatinine while the patients were also tested for noradrenaline and adrenaline determination. 3 patients with essential hypertension, 5 with phaeochromocytoma and 12 with adrenocortical tumours were also submitted to a dynamic test (catecholamine determination before and 2’ after 1 mg glucagon injection) to better define the diagnosis. In patients with phaeochromocytoma, blood samples for catecholamines and CgA were also obtained after surgery (range 15–30 days) in 7 cases (6 benign and 1 malignant).

**Table 1** Demographic and clinical data of normotensive controls (C) and of patients with essential hypertension (EH), phaeochromocytoma (PHEO), other neuroendocrine tumours (other-NET) and with adrenocortical tumours (AT). Mean±SE and range (in parentheses) are reported

| Patients | C | EH | PHEO | other-NET | AT |
|---|---|---|---|---|---|
| No. | 130 | 48 | 20 | 28 | 25 |
| Sex (M/F) | 86/76 | 17/31 | 12/8 | 16/12 | 13/12 |
| Age (yr) | 41.5 ± 2.0 (19–72) | 42.7 ± 1.84 (21–70) | 40.6 ± 3.9 (14–76) | 60.0 ± 3.5# (16–79) | 60.3 ± 1.9# (40–77) |
| SBP (mmHg) | 127.2 ± 1.9**§§° | 148.0 ± 2.6 | 163.5 ± 7.2* | 129.0 ± 2.0**§§° | 153.4 ± 3.7 |
| DBP (mmHg) | 75.9 ± 1.7**§§° | 96.0 ± 1.2 | 102.2 ± 4.7 | 77.5 ± 1.4**§§° | 91.0 ± 2.2 § |
| HR (bpm) | 68.7 ± 1.4 | 70.5 ± 1.2 | 79.8 ± 2.6 | 69.0 ± 1.6 | 69.2 ± 1.9 |
| Pl. Creat. (μM $l^{-1}$) | 77.0 ± 1.7 (44–106) | 79.5 ± 1.7 (53–115) | 73.4 ± 3.5 (38–133) | 76.0 ± 2.6 (44–115) | 95.5 ± 4.4 (53–133) |

M/F: male/female, SBP: systolic blood pressure, DBP: diastolic blood pressure, HR: heart rate, Pl creat: plasma creatinine. * $P < 0.01$; ** $P < 0.001$ vs EH; § $P < 0.02$; §§ $P < 0.001$ vs PHEO; ° $P < 0.001$ vs AT; # $P < 0.0001$ vs C, EH and PHEO.

© 2001 Cancer Research Campaign 

### Assays

Plasma creatinine was measured by a standard technique. Plasma catecholamines and CgA were measured using the same blood sample collected in chilled anticoagulated (EGTA plus L-glutathione reduced for catecholamines; heparin and Trasylol for CgA) glass tubes. Blood was spun down in a refrigerated centrifuge and the plasma stored at −80˚C until the assay. Catecholamines and CgA were then assayed in duplicate and in the same run.

Catecholamines were analysed by high-performance liquid chromatography, as previously described (Krstulovic et al, 1981). The reference range for NA was <400 pg ml$^{-1}$ and for A <80 pg ml$^{-1}$ in supine position.

CgA was measured by using a novel solid-phase two-site immunoradiometric assay based on monoclonal antibodies that bind to two distinct contiguous epitopes within the 145–245 region of CgA (CgA-RIA CT, Cis bio international) (Degorce et al, 1999). The first antibody is coated on the solid phase, the second, used as a tracer, is radiolabelled with iodine 125. CgA present in the standards (recombinant hCgA) is sandwiched between the 2 antibodies. Thus, by using antibodies directed against the median part of the protein, only the region of CgA unexposed to proteolytic processes is involved, allowing detection of the intact form and only the major fragments of the molecule comprising the 145–245 domain.

### Statistical analysis

Variables are expressed as mean, standard error and range. Since in our population, circulating CgA and catecholamines were not normally distributed, analyses were performed using untransformed and log-transformed data. Spearman correlation coefficients were used as parameters of association. The results of hormonal secretion were analysed as positive or negative. Krunskal-Wallis and Wilcoxon tests were performed for each variable to compare groups. *P* levels lower than 0.05 were considered statistically significant.

### RESULTS

Intra-assay and interassay coefficient variations of our CgA method are 2.5% and 7.0%, respectively. Assay detection limit is 1.5 ng ml$^{-1}$ (the smallest detectable concentration different from 0 with a probability of 95%). Values obtained with anticoagulated plasma in 130 normal subjects were: median 44.3 ng ml$^{-1}$; range 21–98.6 ng ml$^{-1}$. A cut-off value was fixed at 100 ng ml$^{-1}$.

As shown in Table 1, controls and patients with essential hypertension and phaeochromocytoma were younger ($P < 0.0001$) than the other 2 groups. Apart from controls, patients with other-NET showed lowest and those with phaeochromocytoma highest blood pressure levels, while blood pressure was similar in patients with essential hypertension and with adrenocortical tumours. No statistical difference in plasma creatinine was found among groups.

CgA levels in essential hypertensives (50.8 ± 3.5 ng ml$^{-1}$) were similar to normotensive controls (49.0 ± 3.1 ng ml$^{-1}$). As shown in Figure 1, where data are log-transformed, CgA in patients with essential hypertension was significantly ($P < 0.0001$) lower than in those with solid cortical adrenal tumours (91.8 ± 13.2 ng ml$^{-1}$), phaeochromocytoma (254.0 ± 49.2 ng ml$^{-1}$) and other-NET (469.5 ± 83.8 ng ml$^{-1}$).

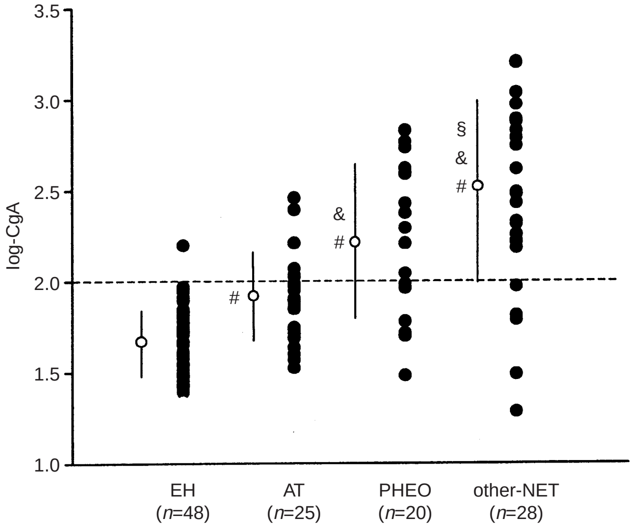

**Figure 1** Plasma log-transformed CgA levels in patients with essential hypertension (EH), adrenocortical tumours (AT), phaeochromocytoma (PHEO) and with other neuroendocrine tumours (other-NET). Individual data and means (±SE) are reported. The dashed line indicates the upper limit of the normal range. # $P < 0.0001$ vs EH; & $P < 0.001$ vs AT; § $P < 0.05$ vs PHEO

Individual data of patients with phaeochromocytoma ($n$ = 20) showed that supine plasma noradrenaline was elevated in 18 (90%), adrenaline in 4 (20%) and CgA in 13 (65%) cases (Figure 2). Thus, we found 2 false negative results for noradrenaline, 16 for adrenaline and 7 for CgA. Noradrenaline and CgA were elevated simultaneously in 11 patients (55%), while adrenaline and CgA in 3 patients (15%). Raised CgA levels associated with normal noradrenaline and adrenaline were found in 2 (10%) and in 10 (50%) cases, respectively. Normal CgA associated with elevated noradrenaline and adrenaline was found in 7 (35%) and in 1 (5%) patients, respectively. In summary, the sensitivity of CgA measurement in identifying patients with phaeochromocytoma was 65%, while that of noradrenaline and of adrenaline was 90% and 20%, respectively. However, CgA measurement in addition to catecholamines improved sensitivity up to 100%.

Log-transformed CgA levels of patients with other-NET ($n$ = 28) are shown in Figure 3. In this group gastroenteropancreatic tumours (607 ± 106 ng ml$^{-1}$) were associated with higher CgA levels than small cell lung tumours (145 ± 42 ng ml$^{-1}$, $P < 0.02$) and medullary thyroid carcinomas (249 ± 209 ng ml$^{-1}$). Individual data showed that in the whole group plasma CgA was greater than normal in 23/28 cases (82%), while in gastroenteropancreatic tumours this finding was observed in all cases (100%). The 5 false negative results were found in 3 out of 6 small cell lung cancers and in 2 out of 3 medullary thyroid carcinomas. CgA and other markers (specific tumoral markers and/or catecholamines) were simultaneously elevated in 17 patients (61%). In 6 cases CgA was found to be the only circulating marker of disease (4 intestinal carcinoids and 2 pancreatic tumours). Thus, sensitivity of the CgA assay in detecting patients with other-NET was 82%, while it was 100% in identifying gastroenteropancreatic tumours. Considering phaeochromocytomas and other-NET as a single group ($n$ = 48), plasma CgA detected the disease in 36 patients (75%), in 8 of whom it was the only circulating marker of disease (17%).

© 2001 Cancer Research Campaign

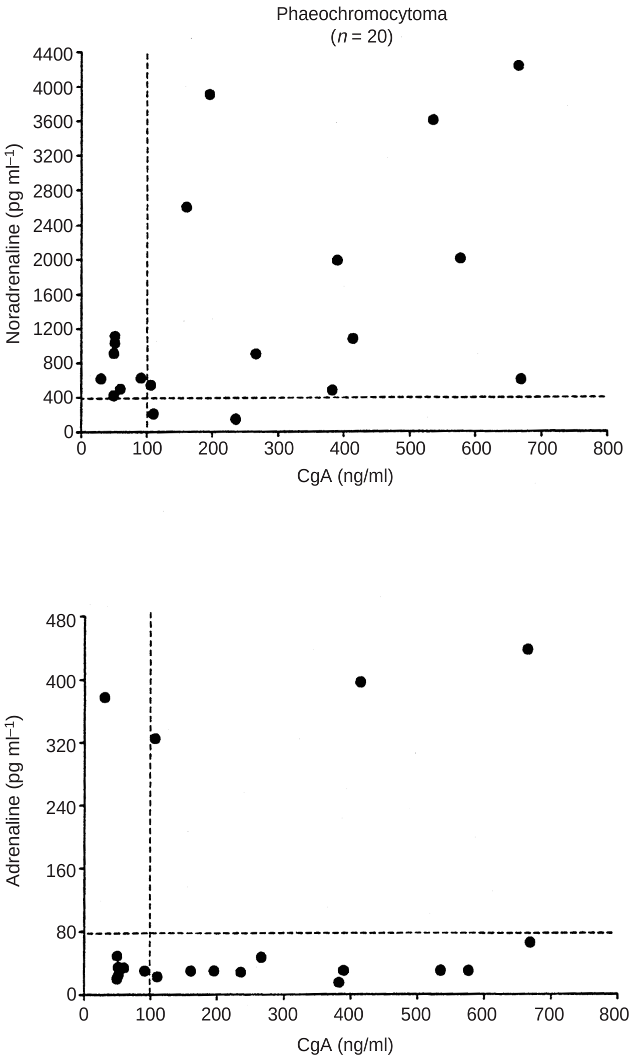

**Figure 2** Plasma supine noradrenaline (above) and adrenaline (below) and CgA levels in patients with phaeochromocytoma. The horizontal dashed lines are the upper normal limit for catecholamines while the vertical dashed lines the upper normal cut-off for CgA levels

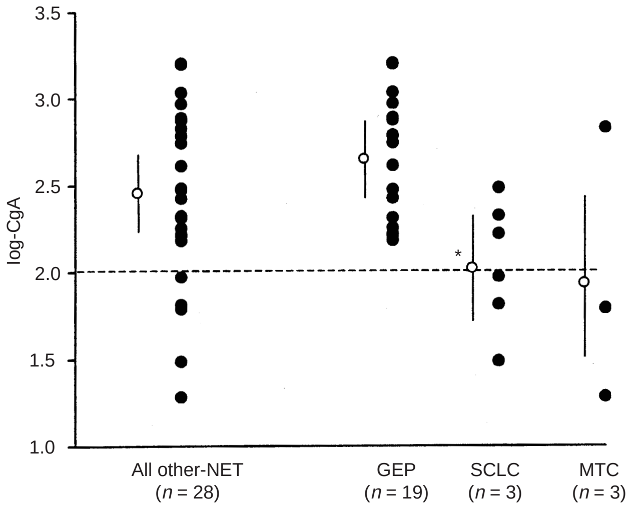

**Figure 3** Plasma log-transformed CgA levels in patients with other neuroendocrine tumours (other-NET) as a whole and subgroups: gastroenteropancreatic (GEP) tumours, small cell lung cancers (SCLC), medullary thyroid carcinomas (MTC). Individual data and means ± (SE) are reported. The dashed line indicates the upper limit of the normal range. *$P$ < 0.02 vs GEP

In patients with adrenocortical tumours ($n$ = 25), supine adrenaline was above the normal range in 1 (4%), noradrenaline in 3 (12%) and CgA in 6 (24%) cases (Figure 4). Plasma noradrenaline and CgA levels were elevated simultaneously in 1 patient (4%), while adrenaline and CgA in no case. Normal CgA associated with elevated noradrenaline and adrenaline was found in 2 (8%) and in 1 (4%) patients, respectively. 17 patients (68%) were negative for both noradrenaline and CgA, and 18 (72%) for both adrenaline and CgA. In the patients with abnormal catecholamines and/or CgA values, diagnosis of phaeochromocytoma was fully excluded in all cases by normal response to the glucagon test, absent metaiodobenzylguanidine-scintigraphy uptake and by radiological features on NMR.

Finally, in essential hypertensives ($n$ = 48), supine plasma adrenaline was in the normal range in all cases, while noradrenaline was elevated in 3 patients (6.2%). Only in 1 case (2.1%) was circulating CgA above normal limits (Figure 5). No patient had catecholamines and CgA simultaneously abnormal. Thus, plasma adrenaline gave no false positive results in patients with essential hypertension, whereas noradrenaline and CgA showed 3 and 1 false positive results, respectively. Consequently CgA specificity was 98%, while that of adrenaline and noradrenaline was 100% and 94%, respectively.

### Associations

Weak positive correlation was found between CgA and plasma noradrenaline in patients with phaeochromocytoma (r = 0.477, $P < 0.05$). No correlation was found between CgA and adrenaline, blood pressure or plasma creatinine either in the group as a whole or in the subgroups. No association was found between tumour size of patients with phaeochromocytoma and plasma CgA or catecholamines.

### Follow-up

All patients with phaeochromocytoma in whom plasma CgA was measured before and after surgery normalized CgA levels, except the patient with recurrence of the disease due to malignant phaeochromocytoma (380 vs 383 ng ml$^{-1}$).

### DISCUSSION

The development of a new method to measure CgA has raised new interest in this protein as a putative circulating marker of neuroendocrine tumours (Degorce et al, 1999). This immunoradiometric assay, based on monoclonal antibodies, makes it possible to bind a region of CgA unexposed to proteolytic processes and, consequently, to detect the intact form and also the major fragments of the molecule. This characteristic offers advantages when CgA is evaluated in pathological conditions where a different proteolysis may generate a variability of the fragments in biological fluids. For example, in patients with phaeochromocytoma, and perhaps with other endocrine tumours, CgA circulates in at least 3 forms

© 2001 Cancer Research Campaign

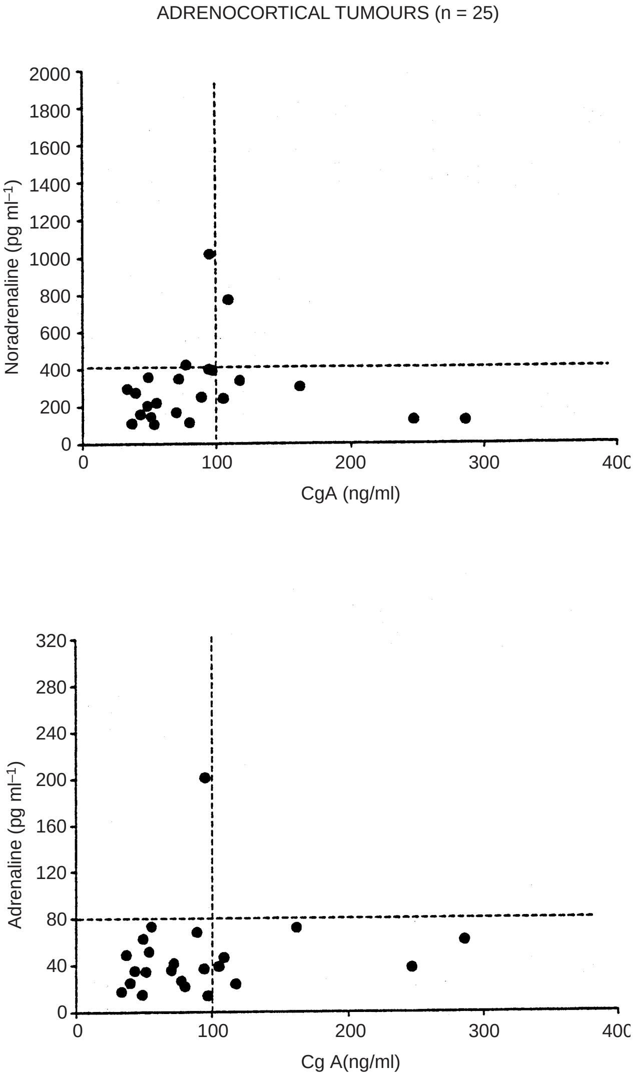

**Figure 4** Plasma supine noradrenaline (above) and adrenaline (below) and CgA levels in patients with solid nonfunctioning adrenocortical tumours. The horizontal dashed lines are the upper normal limit for catecholamines while the vertical dashed lines the upper normal cut-off for CgA levels

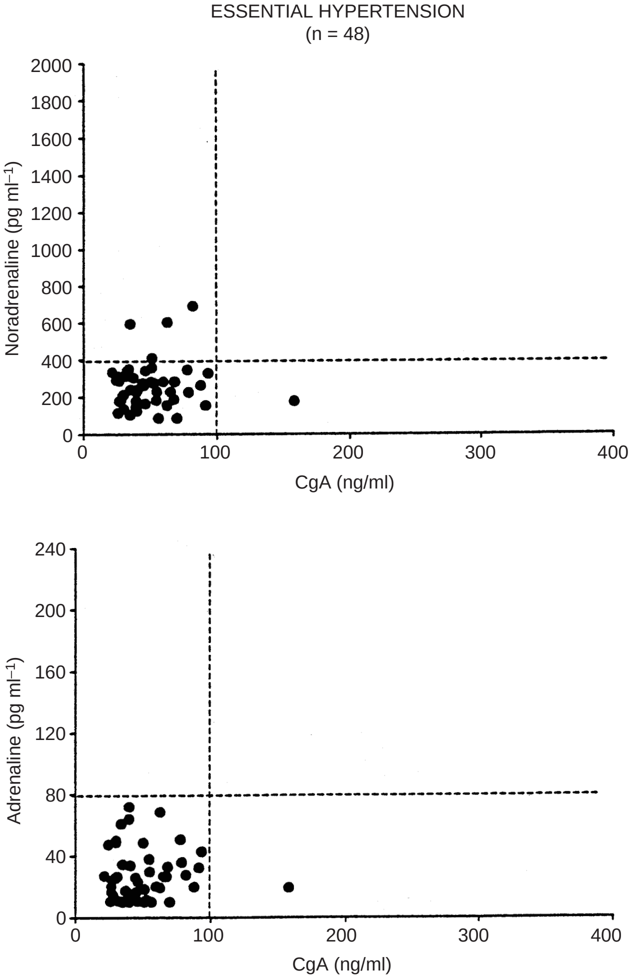

**Figure 5** Plasma supine noradrenaline (above) and adrenaline (below) and CgA levels in patients with essential hypertension. The horizontal dashed lines are the upper normal limit for catecholamines while the vertical dashed lines the upper normal cut-off for CgA levels with similar molecular weights but different immunoreactivity (Corti et al, 1996). Therefore, a method evaluating both intact sequence and major fragments of CgA may be useful in clinical practice.

In the present study we included a large group of patients with phaeochromocytoma. Sensitivity of plasma CgA in detecting this disease reached 65%, a percentage lower than that observed by other authors (Grondal et al, 1991; Canale and Bravo, 1994; Nobels et al, 1997; Stridsberg and Husebye, 1997) who utilized polyclonal radioimmunoassays, but very similar to that of Baudin et al (1998) using the same immunoradiometric method. In addition, our data confirm that phaeochromocytomas usually cosecrete catecholamines and CgA (Grondal et al, 1991; Kimura et al, 1997; Baudin et al, 1998) but also show that occasionally they release only the latter compound. Indeed, in 2 patients with histologically proven phaeochromocytoma, catecholamines were in the normal range while plasma CgA was very high, demonstrating that CgA was the only circulating marker of disease (Grondal et al, 1991; Stridsberg and Husebye, 1997). Therefore, we agree with Stridsberg and Husebye (1997) that measuring of plasma catecholamines in addition to CgA improves diagnostic sensitivity in identifying all true-positive patients, including those with hormone-negative tumours (Sobol et al, 1989). Finally, on the basis of our data, circulating CgA was also found to be useful in following up the patients after surgery. Normalization of plasma CgA occurred in all patients with phaeochromocytoma after removal of the mass, while persistence of high values indicated recurrence of disease in the case of maligant phaeochromocytoma (Grondal et al, 1991; Kimura et al 1997; Stridsberg and Husebye, 1997).

Our study was not planned to compare sensitivity and specificity between CgA and the other tumoral markers in patients with other-NET. However, we confirm that plasma CgA is an excellent predictor of disease, especially in gastroenteropancreatic tumours (O'Connor and Deftos, 1986; Eriksson et al, 1990; Nobels et al, 1997; Baudin et al, 1998) which were identified in all cases. As

*© 2001 Cancer Research Campaign*

already reported for phaeochromocytomas, in this group a frequent cosecretion of CgA and of other tumoral markers was likewise observed (Nobels et al, 1997; Baudin et al, 1998) and CgA was sometimes found to be the only humoral predictor of disease (Baudin et al, 1998; Ferrari et al, 1998; Nobels et al, 1998). Thus, CgA can be used as a serum marker not only for the functioning but also for the so-called 'non-functioning' neuroendocrine tumours. The small number of patients with medullary thyroid carcinoma and small cell lung carcinoma included in the present study does not allow to establish whether CgA, by our method, is adapted when compared to more specific markers such as calcitonin (Blind et al, 1992) and neuron-specific enolase (Akoun et al, 1985), respectively. Therefore, at the present, CgA has not to be considered the first choice marker of these tumours.

In the present paper we included also solid adrenocortical tumours without apparent secretory activity. We unexpectedly found 6 patients with elevated CgA levels, a figure much higher than that observed in essential hypertensives, despite superimposable blood pressure and plasma creatinine values. We have no clear explanation for this finding. However, coexistent unknown tumours, nests of medullary tissue included in the cortical adenoma (Bornstein et al, 1999) or a neuroendocrine differentiation of the adrenal tumour itself (Miettinen, 1992; Li et al, 1998) cannot be excluded to explain the high CgA levels in these patients.

At variance with other reports (Nobels et al, 1997; Baudin et al, 1998), we finally studied an extensive population of patients without tumours, i.e. essential hypertensives. Though previous data (O'Connor, 1985; Hsiao et al, 1991; Canale and Bravo, 1994) indicated that plasma CgA is higher in hypertensives than in normotensives, no information is available with this new method. Our results show that circulating CgA of essential hypertensive patients is similar to that of normotensive controls and almost constantly in the normal range, thus confirming the high specificity of this humoral marker (Hsiao et al, 1991; Canale and Bravo, 1994; Nobels et al, 1997; Baudin et al, 1998).

In conclusion, CgA measurement with this new assay shows good sensitivity in identifying gastroenteropancreatic tumours and, in association with catecholamine determination, in detecting patients with phaeochromocytoma. Furthermore, plasma CgA sometimes appears to be the only humoral marker of disease. Thus the CgA assay, also for its high specificity, may be a precious tool to diagnose both functioning and non-functioning neuroendocrine tumours.

### ACKNOWLEDGEMENTS

We wish to thank Mrs P. Tabucchi and Mister R. Birindelli for their precious technical assistance.

### REFERENCES

Akoun GM, Scarna HM, Milleron BJ, Benichou MP and Herman DP (1985) Serum neuron-specific enolase. A marker of disease extent and response to therapy for small cell lung cancer. *Chest* **87**: 39–43

Barbosa JA, Gill BM, Takiyyuddin MA and O'Connor DT (1991) Chromogranin A: post-translational modifications in secretory granules. *Endocrinology* **128**: 174–190

Baudin E, Gigliotti A, Ducreux M, Ropers J, Comoy E, Sabourin JC, Ridart JM, Cailleux AF, Bonacci R, Ruffie P and Schlumberger M (1998) Neuron-specific enolase and chromogranin A as markers of neuroendocrine tumours. *Br J Cancer* **78**: 1102–1107

Bender H, Maier A, Wiedenmann B, O'Connor DT, Messner K and Schmidt-Gayk H (1992) Immunoluminometric assay of chromogranin A in serum with commercially available reagents. *Clin Chem* **38**: 2267–2272

Blind E, Schmidt-Gayk HS, Sinn H-P, O'Connor DT and Raue F (1992) Chromogranin A as tumor marker in medullary thyroid carcinoma. *Thyroid* **2**: 5–10

Bornstein SR, Sratakis CA and Chrousos GP (1999) Adrenocortical tumours: recent advances in basic concepts and clinical management. *Ann Intern Med* **130**: 759–771

Canale MP and Bravo EL (1994) Diagnostic specificity of serum chromogranin-A for pheochromocytoma in patients with renal dysfunction *J Clin Endocrinol Metab* **78**: 1139–1144

Cetin Y and Grube D (1991) Topology of chromogranins in secretory granules of endocrine cells. *Histochemistry* **96**: 301–310

Corti A, Gasparri A, Chen FX, Pelagi M, Brandazza A, Sidoli A and Siccardi AG (1996) Characterisation of circulating chromogranin A in human cancer patients. *Br J Cancer* **73**: 924–932

Deftos LJ (1991) Chromogranin A: its role in endocrine function and as an endocrine and neuroendocrine tumour marker. *Endocrinol Rev* **12**: 181–187

Deftos LJ, Linnoila RI, Carney DN, Burton DW, Leong SS, O'Connor DT, Murray SS and Gazdar AF (1988) Demonstration of chromogranin A in human neuroendocrine cell lines by immunohistology and immunoassay. *Cancer* **62**: 92–97

Degorce F, Goumon Y, Jacquemart L, Vidaud C, Bellanger L, Pons-Anicet D, Seguin P, Metz-Boutigue MH and Aunis D (1999) A new human chromogranin A (CgA) immunoradiometric assay involving monoclonal antibodies raised against the unprocessed central domain (145–245). *Br J Cancer* **79**: 65–71

Dillen L, De Block J, Van Lear L and Potter W (1989) Enzyme- linked immunosorbent assay for chromogranin A. *Clin Chem* **35**: 1934–1938

Eriksson B, Arnberg H, Oberg K, Hellman U, Lundqvist G, Wernstedt C and Wilander E (1990) A polyclonal antiserum against chromogranin A and B- a new sensitive marker for neuroendocrine tumours. *Acta Endocrinol* (Copenh.) **122**: 145–155

Ferrari L, Seregni E, Martinetti A, Van Graafeiland B, Nerini-Molteni S, Botti C, Artale S, Cresta S and Bombardieri E (1998) Chromogranin A measurement in neuroendocrine tumours. Int *J Biol Markers* **13**: 3–9

Grondal S, Eriksson B, Hamberger B and Theodorsson E (1991) Plasma chromogranin A+B, neuropeptide Y and catecholamines in pheochromocytoma patients. *J Intern Med* **229**: 453–456

Helle K and Angeletti RH (1994) Chromogranin A: multipurpose prohormone? *Acta Physiol Scand* **152**: 1–10

Hsiao RJ, Seeger RC, Yu AL and O'Connor DT (1990a) Chromogranin A in children with neuroblastoma: serum concentration parallels disease stage and predicts survival. *J Clin Invest* **85**: 1555–1559

Hsiao RJ, Neumann HPH, Parmer RJ, Barbosa JA and O'Connor DT (1990b) Chromogranin A in familial pheochromocytoma: diagnostic screening value, prediction of tumour mass, and post-resection kinetics indicating two-compartment distribution. *Am J Med* **88**: 607–612

Hsiao RJ, Parmer RJ, Takiyyuddin MA and O'Connor DT (1991) Chromogranin A storage and secretion: sensitivity and specificity for the diagnosis of pheochromocytoma. *Medicine* **70**: 33–45

JNC VI–World Health Organization–International Society of Hypertension Guidelines for the Management of Hypertension (1999). *J Hypertens* **17**: 151–183

Johnson PWM, Joel SP and Love S (1993) Tumour markers for prediction of survival and monitoring of remission in small cell lung cancer. *Br J Cancer* **67**: 760–766

Kimura N, Miura W, Noshiro T, Mizunashi K, Hanew K, Shimizu K, Watanabe T, Shibukawa S, Sohn HE, Abe K, Miura Y and Nagura H (1997) Plasma chromogranin A in pheochromocytoma, primary hyperparathyroidism and pituitary adenoma in comparison with catecholamine, parathyroid hormone and pituitary hormones. *Endocr J* **44**: 319–327

Konecki DS, Benedurn NM, Gerdes HH and Huttern WB (1987) The primary structure of human chromogranin A and pancreastin. *J Biol Chem* **262**: 17026–17030

Krstulovic AM, Dziedzk SW, Bertan-Dziedzic L and Dirico DE (1981) Plasma catecholamines in hypertension and pheochromocytomas determined using con-pair reversed-phase chromatography with amperometric detection. *J Chromatography* **217**: 523–537

Li Q, Johansson H, Kjellman M and Grimellius L (1998) Neuroendocrine differentiation and nerves in human adrenal cortex and cortical lesions. *APMIS* **106**: 807–817

© 2001 Cancer Research Campaign 

Metz-Boutigue MH, Garcia-Sablone P, Angeletti RH and Annis D (1993) Intracellular and extracellular processing of chromogranin-A. Determination of cleavage sites. *Eur J Biochem* **217**: 247–257

Miettinen M. Neuroendocrine differentiation in adrenocortical carcinoma (1992) Now immunohistochemical findings supported by electron microscopy. *Lab Invest* **66**: 169–174

Nobels FRE, Kwekkeboom DJ, Coopmans W, Shoenmakers CHH, Lindemans J, De Herder WW, Krenning EP, Bouillon R and Lamberts SWJ (1997) Chromogranin A as serum marker for neuroendocrine neoplasia: comparison with neuron-specific enolase and the α-subunit of glicoprotein hormones. *J Clin Endocrinol Metab* **82**: 2622–2628

Nobels FR, Kwekkeboom DJ, Bouillon R and Lamberts SW (1998) Chromogranin A: its clinical value as marker of neuroendocrine tumours. *Eur J Clin Invest* **28**: 431–440

O'Connor DT (1985) Plasma chromogranin A. Initial studies in human hypertension. *Hypertension* **7** [Suppl 1]: I-76–I-79

O'Connor DT and Bernstein KN (1984) Radioimmunoassay of chromogranin A in lasma as a measure of exocytotic sympathoadrenal activity in normal subjects and patients with pheochromocytoma. *N Engl J Med* **311**: 764–770

O'Connor DT and Deftos LJ (1986) Secretion of chromogranin A by peptide producing endocrine neoplasms. *N Engl J Med* **314**: 1145–1151

O'Connor DT, Frigon RP and Sokoloff RL (1984) Human chromogranin A. Purification and characterization from catecholamine storage vescicles of human pheochromocytoma. *Hypertension* **6**: 2–12

Rosa P and Gerdes HH (1994) The granin protein family markers for neuroendocrine cells and tools for the diagnosis of neuroendocrine tumours. *J Endocrinol Invest* **17**: 207–225

Simon JP and Annis D (1989) Biochemistry of the chromogranin A protein family. *Biochem J* **262**: 1–13

Sobol RE, O'Connor DT, Addison J, Suchocki K, Royston I and Deftos LJ (1986) Elevated serum chromogranin A concentrations in small-cell lung carcinoma. *Ann Intern Med* **105**: 698–700

Sobol RE, Memoli V and Deftos LJ (1989) Hormone-negative, chromogranin A-positive endocrine tumours. *N Engl J Med* **320**: 444–447

Stridsberg M and Husebye ES (1997) Chromogranin A and chromogranin B are sensitive circulating markers for pheochromocytoma. *Eur J Endocr* **136**: 67–73

Strub JM, Goumon Y, Lugardon K, Capon C, Lopez M, Moniatte M, Van Dorsselaer A, Aunis D and Metz-Boutigue MH (1996) Antibacterial activity of glycosylated and phosphorylated chromogranin A-derived peptide 173–194 from bovine adrenal medullary chromaffin granules. *J Biol Chem* **271**: 28540–28553

Syversen U, Jacobsen MB, O'Connor DT, Ronning K and Waldum HL (1994) Immunoassay for measurement of chromogranin A and pancreastin-like immunoreactivity in humans: correspondence in patients with neuroendocrine neoplasia. *Neuropeptides* **26**: 201–206

Totsch M, Muller LC, Hittmair A, Ofner D, Gibbs AR and Schmid KW (1992) Immunohistochemical demonstration of chromogranins A and B in neuroendocrine tumours of the lung. *Hum Pathol* **23**: 312–316

Weiler R, Fleichtinger H, Schmid KW, Fischer-Colbrie R, Grimelius L, Cedermark B, Papotti M, Bussolati G and Winkler H (1987) Chromogranin A and B and secretogranin II in bronchial and intestinal carcinoids. *Virchows Arch A* **412**: 103–109

 *© 2001 Cancer Research Campaign*
